# Challenges, Attitudes and Practices of the Spectacle Wearers in a Resource-Limited Economy

**DOI:** 10.4103/0974-9233.61223

**Published:** 2010

**Authors:** Abdulkabir A. Ayanniyi, Feyi G. Adepoju, Rashidat O. Ayanniyi, Regina E. Morgan

**Affiliations:** Department of Ophthalmology, University of Abuja, Nigeria; 1 University of Ilorin, Abuja, Nigeria; 2 Pharmacology and Therapeutics, University of Ilorin, Abuja, Nigeria; 3 National Hospital, Abuja, Nigeria

**Keywords:** Attitudes, Challenges, Positive Change, Practices, Spectacles, Wearers

## Abstract

**Purpose:**

To evaluate challenges, attitudes and practices among spectacle wearers to effect positive change when necessary, and determine positive change in a resource-limited economy.

**Materials and Methods:**

A multi-hospital descriptive, cross sectional survey of spectacle wearers was conducted between May 2007 and December 2008 in Nigeria.

**Results:**

A total of 214 wearers comprising 43.5% males and 56.5% females aged 18-84 years were surveyed. The majority of subjects (92.6%) had at least secondary education. The wearers’ challenges included expensive spectacles (43.0%), falling/scratched/broken lenses (29.4%) and fear that spectacles would damage the eyes (23.8%). The wearers’ attitudes were comprised of consultations with ‘road side dispensers’ (7%) and permitting other individuals to select spectacle frames for them (26%). Care and maintaince practices included use of handkerchief, tissue paper, fingers and water to clean spectacles (49.5%) and placing spectacles inside spectacle cases (30.4%). There were no associations (*P* > 0.05) between gender or literacy levels and who selected the frames for the subjects, caregivers consulted for spectacles, and cleaning materials for spectacles. The placement of spectacles when not in use was significantly associated (*P* < 0.05) with the wearers’ gender and literacy levels but not with the length of spectacle wear.

**Conclusion:**

Attitudes and practices requiring positive change crossed gender and educational levels among spectacle wearers. The cost of spectacles should be regulated and availability of standard eye care practices would reduce challenges including lens-related defects and quackery. During consultation with a recognized eye care professional, counseling of wearers on positive attitudes/practices as well as allaying fear of spectacle wear is required.

## INTRODUCTION

Spectacles or eyeglasses are frames bearing lenses worn in front of the eyes, usually to enhance vision.^1^–^6^ Other reasons for spectacle wear include eye protection, to conceal eyes defects and as a fashion accesory.^5^ Despite the increasing popularity of contact lenses and refractive surgery^7^–^9^, the use of spectacles still remains the most popular method of correcting refractive errors.^1^–^5^

Despite inherent merits such as control over their use, spectacles do pose some significant challenges. For example, spectacles are not readily affordable^1^,^2^,^5^ by many who require them and can be a source of ocular discomfort especially when incorrectly prescribed. Refractive errors are a major cause of visual impairment globally.^10^ There has been abuse of spectacle dispensing due to a lack of standardization, superfluous prescriptions and distribution by individuals who have no professional experience in eye care or dispensing.^11^,^12^

Although many studies had been conducted on refractive errors^11^,^13^–^17^ there comparatively few studies on attitudes and practices among spectacle wearers. The aim of this study was to provide knowledge to bridge this important gap. The findings in this study are important and will assist stakeholders in the eye care industry to enhance eye care.

## MATERIALS AND METHODS

This was a prospective, cross-sectional study conducted between May 2007 and December 2008 at six health facilities in Nigeria. The six health facilities were located in the cities of Ilorin and Ado Ekiti, both state capitals in Nigeria.

The participants in this study were spectacle wearers who presented for eye consultations in the eye clinics of the six health care facilities. The criteria for inclusion in the study were age 18 years or older, current use of spectacles for a period of at least six months, and consent to participate in the study. Informed consent was obtained from all subjects and the study was performed in accordance with the tenets of the Declaration of Helsinki. Each subject was interviewed using a prepared questionnaire. The questionnaire had been previously pretested at one of the health facilities in Ilorin, on spectacle wearers who were not included in the analysis. The questionnaire covered the demographic parameters, challenges, attitudes and practices on the use of spectacles.

The data were entered into SPSS 15.0 (SPSS Inc., Chicago, IL, USA), and analyzed. The associations were tested using the *Chi* square test and was considered significant at a level of *P* < 0.05.

## RESULTS

Two hundred and fourteen spectacle wearers comprised of 93 (43.5%) males and 121 (56.5%) females were surveyed. The mean age of the study cohort was 40.2 ± 15.8 years (range,18 years to 84 years). Cohort demographics are presented in Table 1. All subjects wore spectacles for at least six months and 194 (90.7%) subjects found the spectacles were satisfactory or very satisfactory for their needs [Table 2].

**Table 1:** Demographic data of spectacle wearers

Age	Gender		Vocation n = 214						
		
	M	F	*CS	Student	Teacher	Pensioner	Trader	Artisan	Others
<20	6	10		15	1				
21-30	24	37	11	41	2		1	3	4
31-40	13	14	10	1	3		3	5	5
41-50	26	26	31		9		7		3
51-60	8	25	12		11	3	5	1	3
61-70	12	7				11	2	3	
71-80	3	2				6			
>80	1								
Total	93	121	66	57	26	20	18	12	15
	Marital status				Educational level				
			
	Married	Single	Widow	Widower	Tertiary	Secondary	Primary	†NFE	

No.	139	70	4	1	151	47	9	7	
%	65.0	32.7	1.9	0.5	70.6	22.0	4.2	3.3	

* CS denotes civil servant,

† NFE denotes No formal education, No denotes number of subjects

**Table 2:** Duration of spectacle use and satisfaction with current spectacles

	No. (n = 214)	%
Duration of spectacle use		
6 months to <1 year	55	25.7
1-5 year	72	33.6
>5-10 years	40	18.7
>10 years	47	22.0
Rating of spectacle usefulness		
Very satisfactory	86	40.2
Satisfactory	108	50.5
Unsatisfactory	15	7.0
Very unsatisfactory	5	2.3

No denotes number of subjects

**Figure 1:**
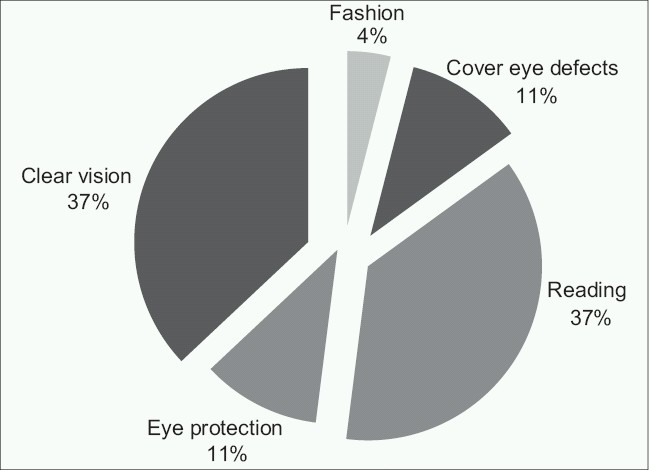
Reasons for spectacle wear

The most frequent reasons for spectacle wear were reading in 80 (37.4%) subjects and distance vision in 80 (37.4%) subjects [Figure 1]. There were a number of challenges to spectacle wear with the high cost of the spectacles being the most common [Table 3].

**Table 3:** Challenges to spectacle use identified by spectacle wearers

Challenges	No. (n = 214)	%
Costly spectacles	92	43.0
Falling/scratch/broken lenses	63	29.4
Fear of spectacles damaging the eyes	51	23.8
Distorted vision	40	18.7
The frames leave an impression on the face	32	15.0
Incorrect prescription	23	10.7
Heavy spectacles	16	7.5
Quackery in eye care industry	09	4.2

There were multiple entries by some wearers; No denotes number of subjects; % denotes percent of subjects

**Figure 2:**
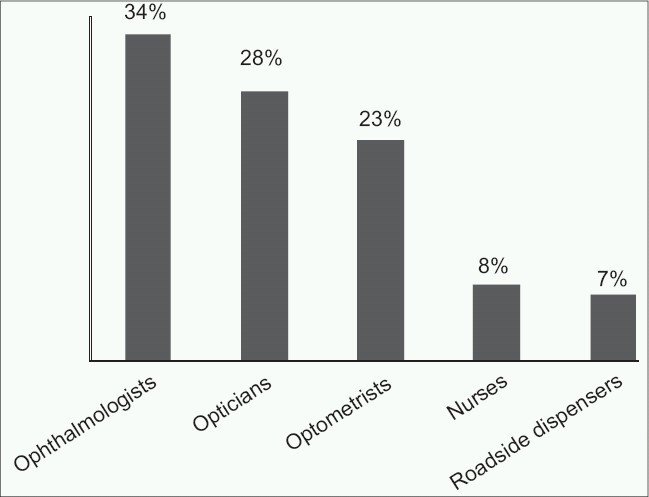
Distribution of wearers by caregivers consulted for spectacles

Subjects consulted a variety of providers to buy spectacles including ‘road side dispensers’ 16 (7%) [Figure 2]. In 26.2% of the cases, spectacle frames were not selected by the wearers [Table 4]. Almost half of the cohort (49.5%) did not use a lens cleaner to clean their spectacles [Table 5].

**Table 4:** Attitude of spectacle wearers to selecting spectacle frames

Who picked frames for wearers	No. (n = 214)	%
Wearers themselves	158	73.8
Spectacle sellers	31	14.5
Partners	14	6.6
Parents	11	5.1

No denotes number of subjects; % denotes percent of subjects

**Table 5:** Maintenance and storage of spectacles

Practice	No. (n = 214)	%
Materials frequently used to clean spectacles		
Lens cleaner	108	50.5
Handkerchief	59	27.6
Tissue paper	23	10.5
Any available material	18	8.4
Water	4	1.9
Fingers	2	0.9
Places where spectacles are frequently kept		
Inside spectacle case	150	69.6
Any available space	29	13.6
Inside bag	19	8.9
On table	17	7.9

No denotes number of subjects; % denotes percent of subjects

Spectacles were not stored in their cases by 30.4% of the cohort [Table 5].

There were no associations between subject gender and the individual who selected their frames (*P* = 0.234), or the caregivers whom the subjects consulted for spectacles (*P* = 0.055), or the cleaning materials used (*P* = 0.732). There were no associations between the subjects’ educational level and who selected the frames for them (*P* = 0.216), or the caregivers whom they consulted for spectacles (*P* = 0.129), or the cleaning regimen (*P* = 0.372). There was no assocation in the duration of spectacles use and how the spectacles were stored (*P* = 0.141). Gender (*P* = 0.006) and educational levels (*P* = 0.029) were significantly associated with how the spectacles were stored.

## DISCUSSION

This study found a significant association between gender and the method used by subjects to store their spectacles. However, the gender was skewed in this study with females being 1.3 times greater than the number of males. Analyses found that gender had no association with who selected the frames for the subjects.

The two most common age groups (21-30 years; 41-50 years) using spectacles correlated with vocations such as civil service and school attendance, due to the need for clear vision. Subjects in the 41-50 years age category required presbyopic spectacles to function effectively and perform at an optimal level.

Over 90% of the study cohort had at least secondary education which was commensurate with the two most common reasons for spectacle wear (reading and clear vision). Similar to previous studies^18^–^21^ that underscore the positive influence of education on health conditions, we found the education level was significantly associated with the way subjects frequently stored their spectacles. Remarkably, higher educational levels were not associated to who selected the spectacle frames for the subjects. It was observed that the wearers consulted the caregivers on the choice of spectacles, and the cleaning materials required for the spectacles.

In this cohort, 75% of the subjects had worn spectacles for at least a year and the remaining individuals for at least six months. This duration of wear makes their views representative of the spectacle wearing population. However, we found, the duration of spectacles use had no relationship with how the spectacles were frequently stored.

In this study, the over whelming majority of the cohort (90.2%) confirmed the utility of spectacles. This was likely due to the fact that spectacle wear allowed individuals to see well. Undoubtedly, the use of spectacles can improve the quality of life of many individuals who have uncorrected refractive errors.^2^ Visual impairment due to uncorrected refractive error is a global health burden. However, this is a treatable form of blindness with a positive effect on quality of life when corrected. The effective use of spectacles in correcting visual impairment and blindness from refractive errors will improve quality of life by enhancing educational opportunities among children and by inclusion of adults in productive working life.^13^ Other significant effects include the reduction of morbidity from hip fractures and falls among the elderly.^22^

Notably, most subjects consulted eye care professionals for their spectacles. The subjects who were displeased with the use of spectacles should be those who had significant challenges using spectacles. The consultation of ‘road side dispensers’ suggested ignorance on the part of some subjects. It was not unusual for some spectacle wearers to circumvent professional services by consulting quacks for a number of reasons including the ‘false belief’ of cost savings.

Important challenges were expressed by spectacles wearers in this study. Many wearers identified the cost of spectacles as an important issue. In Tanzania, a study identified cost of spectacles as one of barriers to spectacles use by secondary school students.^23^ Other problems including falling/scratched/broken lenses could be reduced with consumer education and by the spectacle dispensers. For example, the providers should make available affordable, quality spectacles while the wearers should seek spectacles from specialists and practice proper care and maintenance of their spectacles. The challenges of distorted vision and incorrect prescriptions could be minimised by obtaining spectacles from eyecare professionals. This could also reduce the incidence of quackery, providing a ‘double barrel’ solution, as quackery could only thrive if there is demand.

The fear of spectacles damaging the eyes although unfounded, was a significant hinderance to spectacle use and is not limited to this report. In studies of Chinese children, a common reason for not wearing spectacles was the belief that spectacles weaken the eyes.^24^,^25^ In a report among secondary school students in Tanzania, this ‘fear’ was referred to as ‘parental concerns about safety of spectacle use’ and listed among the barriers to use of spectacles by students.^23^ It has been suggested that spectacle wear could disrupt normal ‘emmetropization’ (which depends on growth of the eye, the refractive state, and visual stimulation) in infants. Yet, the long term effect of spectacles on normal changes in the refractive error of the human eye is negligible.^6^ The fear of spectacles damaging the eyes should be directly addressed and the concerns alleviated during consultations at eye care clinics.

The 7.5% of subjects who reported the weight of spectacles as a challenge to spectacle wear could have been aphakes or high myopes or hyperopes. Apart from the unfounded fear of ocular damage due to spectacle wear, other challenges as expressed by the wearers were genuine and should be addressed by stakeholders in eye care. Appropriate health education of wearers on care for spectacles should be provided at eye care clinics. The eye care providers should ensure that effective and affordable spectacles are made available to the spectacle wearing population. Self selection of frames may reduce dissatisfaction with frames that may otherwise be selected by other individuals who may have the same tastes. The practice of cleaning spectacles with lens cleaner and storing them in their cases was acceptable and should be reinforced at consultations. Additionally educating wearers on avoiding the use of materials that are not recommended for cleaning their lenses and proper storing and handling would enhance the longevity of spectacles.

One caveat remains, a resource-limited economy has an inadequate number of eye care providers. The ophthalmologists and optometrists who are the key players in managing un-corrected refractive errors are inadequate in number and are available mainly in major cities. This lack of availability and inadequate distribution results in the majority of individuals with refractive error being underserviced or neglected.^26^ However other eye care professionals such as refractionists, opticians and ophthalmic technicians with limited training but relevant skills should be available to provide basic services for the correction of refractive error including presbyopia correction at primary and secondary eye care clinics. This solution would result in the majority of the population being effectively served.^27^,^28^ We recommend that refractionists, opticians and ophthalmic technicians update their knowledge and skills through periodic retraining. The availability of adequately trained eye care providers would reduce incidence of spectacle intolerance from improperly fitted lenses, distorted vision, incorrect prescriptions and quackery.

In conclusion, attitudes and practices requiring positive change cut across gender and educational levels among spectacle wearers. One possible solution is regulation of the cost of spectacles. Availability of qualified eye care providers in sufficient numbers can ensure standard eye care practices that would reduce spectacle-associated challenges including lens related defects and quackery. Spectacle wearer and prospective candidates should be educated on spectacles wear, emphasizing positive attitudes and practices, and eliminating unfounded fear of spectacles during consultations.
